# Supplementary material for: Identification of photoperiod-induced specific miRNAs in the adrenal glands of Sunite sheep (Ovis aries)
Source: Front Vet Sci. 2022 Jul 22;9:888207. doi: 10.3389/fvets.2022.888207 (PMC9354845; doi:10.3389/fvets.2022.888207)
Supplement: Supplementary file 1 [file Data_Sheet_1.ZIP › Supplementary file/miRDeep2 file.zip!/miRDeep2 file/Know_Structure/LP42A1/oar-mir-17.pdf]

miRBase precursor : oar-mir-17
Total read count : 4936
oar-miR-17-5p read count : 4883
remaining reads : 53

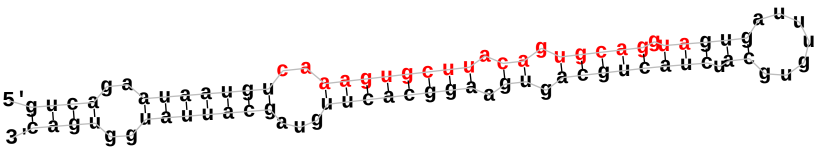

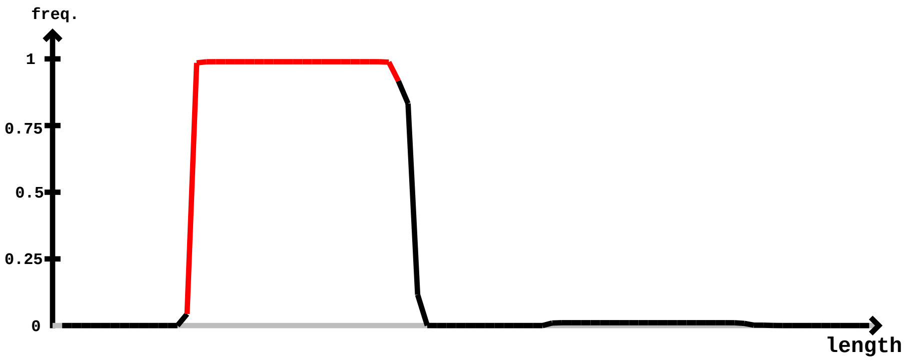


```
                oar-miR-17-5p
5'- gucagaauaaugucaaagugcuuacagugcagguagugauuugugcaucuacugcagugaaggcacuuguagcauuauggugac  -3'    exp
    ((((..(((((((..((((((((.((.(((((.(((((.......)).))))))))).)).))))))))...)))))))..))))   reads     mm        sample
    ............ucaaagugcuuacagugcagg...................................................   3         0         seq
    ............ucaaagugcuuacagugcaggu..................................................   43        0         seq
    ............ucaaagugcuuacagugcaggua.................................................   43        0         seq
    ............ucaaagugcuuacagugcagguag................................................   117       0         seq
    ............ucaaagugcuuacagugcagguagu...............................................   8         0         seq
    .............caaagugcuuacagugcagg...................................................   4         0         seq
    .............caaagugcuCacagugcaggu..................................................   1         1         seq
    .............caaagugcuuacagugcaggu..................................................   305       0         seq
    .............caaagugcuuacagugcaggua.................................................   369       0         seq
    .............caaagugcuCacagugcaggua.................................................   1         1         seq
    .............caaagugcuCacagugcagguag................................................   20        1         seq
    .............caaagugcuuacagugcagguag................................................   3385      0         seq
    .............caaagugcuCacagugcagguagu...............................................   1         1         seq
    .............caaagugcuuacagugcagguagu...............................................   561       0         seq
    .............caaagugcuuacagugcagguagug..............................................   1         0         seq
    ..............aaagugcuuaUagugcaggu..................................................   1         1         seq
    ..............aaagugcuuacagugcaggua.................................................   3         0         seq
    ..............aaagugcuuaUagugcagguag................................................   2         1         seq
    ..............aaagugcuuacagugcagguag................................................   14        0         seq
    ...............aagugcuuaUagugcagguag................................................   1         1         seq
    ..................................................acugcagugaaggcacuug...............   1         0         seq
    ..................................................acugcagugaaggcacuugu..............   12        0         seq
    ..................................................acugcagugaaggcacuugua.............   31        0         seq
    ..................................................acugcagugaaggcacuuguag............   1         0         seq
    ..................................................acugcagugaaggcacuuguagc...........   1         0         seq
    ..................................................acugcagugaaggcacuuguagca..........   1         0         seq
    ...................................................cugcagugaaggcacuugua.............   1         0         seq
    ...................................................cugcagugaaggcacuuguagc...........   4         0         seq
    ...................................................cugcagugaaggcacuuguagca..........   1         0         seq
```
